# Determinants of adherence to nutrition-related cancer prevention guidelines among African American breast cancer survivors

**DOI:** 10.21633/jgpha.6.2s06

**Published:** 2016

**Authors:** Lindsey A. Ramirez, Yunmi Chung, Yoo Wonsuk, Brittney Fontenot, Benjamin E. Ansa, Mary S. Whitehead, Selina A. Smith

**Affiliations:** 1 Master of Science in Psychology, Augusta University, Augusta, GA; 2 Institute of Public & Preventive Health, Augusta University, Augusta, GA; 3 Dental College of Georgia, Augusta University, Augusta, GA; 4 SISTAH Talk Breast Cancer Support Group, Miami, FL; 5 Department of Family Medicine, Medical College of Georgia, Augusta University, Augusta, GA

**Keywords:** African Americans, breast cancer survivors, nutrition guidelines, adherence, health- related quality of life

## Abstract

**Background:**

Mortality rate for breast cancer is higher among African American (AA) women than for women of other racial/ethnic groups. Obesity, also higher among AA women, may increase the risk of breast cancer development and recurrence. Lifestyle factors such as healthy nutrition can reduce the rate of obesity and breast cancer. This study examined the determinants of adherence to nutrition-related cancer prevention guidelines among AA breast cancer survivors.

**Methods:**

AA breast cancer survivors (n=240) were recruited from a breast cancer support group to complete a lifestyle assessment tool for this cross-sectional study. Chi-square test and ordinal logistic regression analysis were used to examine the relationship between adherence to nutrition-related cancer prevention guidelines and potential predictors of adherence.

**Results:**

Majority of the survivors met the guideline for red and processed meat (n=191, 83.4%), but did not meet the guideline for fruits and vegetables (n=189, 80.4%). For survivors with annual household incomes < $25,000, the odds of meeting or partially meeting the guideline for fruits and vegetables was 75.4% less than for participants with incomes > $50,000 (OR= 0.25, 95% CI: 0.08, 0.80). Poor physical functioning (OR= 38.48, 95% CI: 2.26, 656.58), sleep disturbances (OR= 60.84, 95% CI: 1.61, 2296.02), and income > $50,000 (OR= 51.02, 95% CI: 1.13, 2311.70) were associated with meeting the guideline for red and processed meat.

**Conclusions:**

Many AA breast cancer survivors are not meeting the nutrition-related cancer prevention guidelines. For this population, more interventions that enhance access to and consumption of healthy diets are needed.

## INTRODUCTION

Breast cancer is prevalent among African American (AA) women and for this population, the second leading cause of cancer-related mortality (American Cancer Society (ACS), 2016). Racial-ethnic disparities have emerged for women diagnosed with breast cancer; relative to white women, AA women have lower incidence rates but a 42% higher mortality rate (DeSantis et al., 2016). Although breast cancer mortality has been decreasing since 1990, the decline is less for AA women than for white women, accentuating the racial-ethnic disparity and stressing the importance of working with this population (DeSantis et al., 2016).

The National Health and Nutrition Examination Study (NHANES) analyzed trends in obesity between 1999 and 2010, and during this time, obesity in AA women increased (Flegal et al., 2012). Obesity may increase risk of developing cancer and cancer recurrence (Kushi et al., 2012; Smith et al., 2015). Protani et al. (2010) found that breast cancer survivors (BCSs) who were obese had worse survival rates than those who were not obese. Monitoring dietary intake is especially important for AA BCSs due to their increased risk of obesity (Smith et al., 2015). The ACS guidelines are intended to help in maintaining a healthy weight, reducing cancer recurrence, and increasing survival. It is recommended that BCSs consume at least 2.5 cups (5 servings) of vegetables and fruits daily, select whole grains instead of refined grains, and limit consumption of red meat and processed meat (Kushi et al., 2012).

Factors that may influence diet include health-related quality of life (HR-QoL), age, employment, education, income, and marital status (Smith et al., 2015). HR-QoL measures include anxiety, depression, fatigue, and pain intensity. Obesity correlates with a lower HR-QoL, which may influence survival outcomes (Cohen et al., 2016; Andersen, 2002) and there is an association between diet and HR-QoL (Milte et al., 2015; Cohen et al., 2016; Song et al., 2015). Adults over the age of 50 are at a greater risk of eating an unhealthy diet and of developing cancer (ACS, 2016). Time and money are barriers to healthy eating (Macdiarmid et al. 2013). Individuals’ daily schedules, such as going to work, may be a barrier to preparing healthy meals. Additionally, single and high-income earners are more likely to consume convenience food (Lee & Lin 2012). Persons who have a higher education and live with a spouse or children are likely to consume healthier diets (Skuland 2015).

The present investigation sought to determine, for a sample of AA BCSs, the factors that predict adherence to nutrition-related cancer prevention guidelines. Although previous studies have used diet as a predictor of HR-QoL (Blanchard et al., 2008), we examined a bi-directional effect.

## METHODS

### Participants

Following IRB approval from the Morehouse School of Medicine, 240 BCSs were recruited for the study by convenience sampling from Survivors Involving Supporters to Take Action in Advancing Health (SISTAAH) Talk, a BCS support group. Following consent, survivors completed a lifestyle assessment tool (LAT), and data were collected from 2013 to 2015.

### Procedures

The 30-minute LAT was completed self-administered via email or postal mail; or facilitator-administered in-person or by telephone. The questionnaire consisted of demographic factors, breast cancer diagnosis and treatment history; HR-QoL; weight history; physical activity; dietary intake; overall health; and breast cancer knowledge, attitudes, and beliefs. The present report utilized the HR-QoL and dietary intake components of the LAT.

### Outcome Variables

The dietary intake section of the LAT consisted of 25 items. Participants indicated consumption frequencies of various food items per month in terms of days or weeks. The dietary intake section was divided into categories relating to the ACS dietary guidelines of fruits and vegetables and red and processed meat. The ACS recommends 5 daily servings of fruits and vegetables (Kushi et al., 2012), which was designated as the “meeting” category. This value was halved to set the cut-off for “partially meeting”; and value below this was classified as “not meeting.” The final cut-offs for fruit and vegetable daily servings were: meeting=5, partially meeting=2.5–4.99, and not meeting=0–2.49.

The present report utilized McCullough et al.’s (2011) equation to calculate the percentage of whole grains consumed: daily servings of whole grains/(daily servings of whole grains + daily servings of refined grains).

The World Cancer Research Fund International (WCRFI) defined limited intake of red meat as less than 18 ounces a week (WCRFI, n.d.). Ounces were changed into daily servings using the conversion: 14oz= 5 servings (WCRFI, n.d.), which resulted in 6.4 servings per week. “Meeting” was set as 6.39 servings a week and this was doubled to create the cut-off for “partially meeting.” The final cut-offs for red and processed meat daily servings were: meeting=0–0.91, partially meeting=0.92–1.82, and not meeting≥1.83.

Open-ended responses for type of cereal were classified as whole or processed grains according to their ingredients. Cereals with “whole grain” on the nutritional label were categorized as whole grain; cereals lacking this ingredient were categorized as processed (refined) grains. The following values were assigned: 1=whole grains and 2=processed grains. Multiple responses from one participant were each assigned a score. A final score of 1 or 2 was assigned depending on the category with more cereals (i.e., a response of 1, 1, and 2 was coded as 1.) A value of 2 was assigned when the number for cereal in each category was equal (i.e., a response of 1 and 2 was coded as 2).

### Independent Variables

HR-QoL was measured through the Patient Reported Outcomes Measurement Information System (PROMIS), an assessment measuring survivors’ subjective physical, emotional, social, and cognitive functioning in the context of their breast cancer symptoms and treatment. PROMIS has constructed item banks (a collection of questions measuring the same thing that can be administered in short forms or adaptively through computerized adaptive testing). Short forms require 4–10 items; computerized adaptive testing require 3–7 items for more precise scores. PROMIS item banks and their short forms provide evidence that they are reliable and precise measures of generic symptoms and functional reports comparable to legacy instruments (Cella et al. 2010). The HR-QoL section of the LAT consisted of 27 items divided into 8 sub-categories, namely, physical functioning, anxiety, depression, fatigue, sleep disturbance, satisfaction with social role, pain interference, and pain intensity. Cronbach’s alpha was 0.74. All items were scored on a Likert-type scale. Participants rated their physical functioning on a scale of 1=unable to do to 5=without any difficulty; anxiety and depression on a scale of 1=never to 5=always; fatigue, satisfaction with social role, and pain interference on a scale of 1=not at all to 5=very much; sleep disturbance on a scale of 1=very good to 5=very poor; and pain intensity on a scale of 0=no pain to 10=worst imaginable pain. Each HR-QoL section was divided into Good (1, 2, and 3) and Poor (4, 5). Physical functioning, sleep disturbance, and satisfaction with social role were reverse-scored. Pain intensity was converted into a 5-point scale by halving all responses and assigning a number of 1 through 5 in the following manner: 0–1=1, 1.5–2=2, 2.5–3=3, 3.5–4=4, and 4.5–5=5.

Demographic variables included age, education, employment, income, and marital status. Breast Cancer Diagnosis and Treatment variables were hormone receptor status, recurrence, surgery, chemotherapy, radiation, hormone treatment, bone marrow/stem cell transplant and years since diagnosis. Breast Cancer Diagnosis and Treatment History was captured through the NHIS Cancer Control Supplement (National Health Interview Survey 2009–2010) questions focused on issues pertaining to knowledge, attitudes, and practices in cancer-related health behaviors, screening, and risk assessment. Body mass index (BMI) and post-diagnosis weight gain were variables for obesity. BMI was calculated by using height and weight data. Weight history was determined based on responses to the National Health and Nutrition Examination Survey (NHANES) (National Health and Nutrition Examination survey 2009– 2010), a national questionnaire assessing the health and nutritional status of adults and children in the US. The World Health Organization defines obesity as a BMI > 30kg/m^2^ (James et al., 2015). The validity and reliability of the NHIS and NHANES surveys are generally high; and are similar to those of the BRFSS, since they all produced similar estimates for several outcome measures, and many of the observed differences were found to have limited consequences for implementing related public health programs (Fahimi et al. 2008).

### Statistical Analyses

Participant characteristics were presented as frequencies and percentages for categorical variables and as means ± standard deviation (SD) for continuous variables. To compare participant characteristics across the “fruit and vegetable” and “red and processed meat” outcome categories, chi-square tests were used. To identify factors associated with meeting the dietary guidelines, multivariable logistic regression modeling with purposeful selection of covariates were used with a p-value cut-off of 0.25 (Bursac et al., 2008). All demographic variables (age, education, employment, income, and marital status) were included in the model regardless of their significance level. The odds ratios and corresponding 95% confidence intervals (CI) were reported from fitted logistic regression model. Multicollinearities among selected factors and demographic variables were examined in order not to avoid overestimation of variance and underestimation of tests (Kleinbaum et al., 2008; Yoo et al., 2014). Statistical analyses were performed using IBM SPSS Statistics version 21. All tests were two-tailed, and p-values less than 0.05 were considered statistically significant.

## RESULTS

Majority (91.3%) of the 240 participants were African American/black, between 50–64 years of age (49.2%), married (40.8%), and had completed some college education or higher (71.7%) (Table 1). Half (50.0%) of the participants were employed, and 36.7% had annual household income between $25,000 and $49,999. Almost 84% of the participants met the guideline for red and processed meat; however, 80.4% did not meet the guideline for fruit and vegetable consumption.

### Meeting the guideline for fruit and vegetable consumption

The proportion of participants who met the guideline for fruit and vegetable consumption was small (n=4, 1.7%) (Table 1).

Bivariate analyses of demographic, clinical, body weight, and HR-QoL variables, with the outcome variable of meeting the guideline for fruit and vegetable consumption guidelines (meet/partially meet/not meet) showed no statistically significant differences among the outcome groups in participant characteristics (Table 2).

Regardless of the outcome group, most participants (45.7%) reported being diagnosed with breast cancer 5–10 years duration without recurrence (80.3%), and had received surgery (85.3%) and chemotherapy (54.5%). Most (69.1%) had not received hormone treatments or a bone marrow/stem cell transplant (97.9%). Only about a third (30%) of the participants were in the healthy weight category with BMI values less than 25 kg/m^2^; and the remaining 70% were either overweight or obese. Almost 55% reported gaining approximately 20lbs or more post-diagnosis. Relative to anxiety, depression, fatigue, sleep disturbance, satisfaction with social role, pain interference, and pain intensity, most participants had “good” HR-QoL scores. For most participants, however, their physical functioning score was “poor”.

### Meeting the guideline for consumption of red and processed meat

Contrary to the results for fruit and vegetable consumption, most of the participants met the guideline for consumption of red and processed meat, with only 3.3% (n=8) not meeting the guideline (Table 1). Regardless of the meat consumption group, most participants were between 50–64 years of age and were employed (Table 3).

In Table 3, meeting the guideline for consumption of red and processed meat was significantly associated with higher educational attainment (p=0.048); 76.7% of participants who met the guideline had at least some college level education, while 23.3% of those not meeting the guideline had less than college education. Marital status, annual household income, and pain intensity were also significantly associated with meeting the guideline for consumption of red and processed meat (p=0.040, p=0.026, and p=0.049 respectively). The proportion of participants meeting this guideline was the lowest among singles (18.9%), among those with annual household incomes less than $25,000 (25.3%), and those reporting “poor” pain intensity (47.3%).

Although the association for meat consumption and body weight or clinical characteristics were not statistically significant (Table 3), most of the participants reported being 5–10 years post breast cancer diagnosis, and with no recurrence. Majority reported receiving surgery to remove tumors and receiving chemotherapy as part of their treatment. Regardless of the outcome category, most of the participants were in the overweight or obese category. Although the proportion of participants who gained more than 20lbs post-diagnosis was lower among those who met the guideline compared to those who partially met the guideline, the overall difference was not significant (p=0.228).

Similar to the patterns for fruit and vegetable consumption, regardless of meat consumption, most participants had good HR-QoL scores in terms of anxiety, depression, fatigue, sleep disturbance, satisfaction with social role, and pain interference, whereas their physical functioning score was poor. The proportion of participants who had good pain intensity scores was highest among those that partially met the guideline for consumption of red and processed meat guideline (76.7%) compared to those that met (52.7%) or did not meet the guideline (57.1%).

### Regression analysis of factors related to meeting dietary intake guidelines

In the ordinal regression model for fruit and vegetable consumption, five demographic variables (age, education, employment, income, and marital status) and one clinical characteristic variable (surgery) were included. The result of the ordinal logistic regression showed an association between meeting/partially meeting fruit and vegetable guidelines and annual household income (Table 4).

Overall, participants who earned less than $50,000 per year were less likely to meet or partially meet the fruit and vegetable guideline than those who made $50,000 or more per year. Among participants making less than $25,000, the odds of meeting or partially meeting the fruit and vegetable guideline were 0.246 (95% CI: 0.075, 0.801; p=0.020) compared to those making more than $50,000. The odds of meeting or partially meeting the fruit and vegetable guideline among participants making between $25,000 and $49,999 was 0.318 (95% CI: 0.131, 0.774; p=0.012) compared to those making more than $50,000.

In the ordinal regression model for consumption of red and processed meat, five demographic variables, four body weight/clinical characteristics (year since diagnosis, breast cancer recurrence, BMI, and post-diagnosis weight gain), and three HR-QoL factors (physical functioning, sleep disturbance, and pain intensity) were included. Results of the ordinal logistic regression revealed that poor physical functioning scores (OR=38.481 (95% CI: 2.255, 656.579; p=0.012)) and poor sleep disturbance score (OR= 60.841 (95% CI: 1.612, 2296.022; p=0.027)) were associated with meeting guidelines for consumption of red and processed meat, and lower annual household income was associated with partially meeting or not meeting the guidelines (Table 5).

Among participants making less than < $25,000, the odds of meeting red and processed meat guideline were 0.020 (95% CI: 0.000, 0.888, p=0.043) compared to those making more than $50,000 (Table 5). The odds of meeting the guideline among participants making between $25,000 and $49,999 was 0.008 (95% CI: 0.000–0.513, p=0.023) compared to those making more than $50,000.

## DISCUSSION

The current study examined, for a sample of AA BCSs, socio-demographic, medical and HRQoL factors associated with adherence to dietary guidelines. Overall, for the combined dietary guidelines, most of the participants were not meeting or were partially meeting all of the recommendations. The results however show that most of the participants (80%) were meeting the recommended intake of red/processed meat, but not for fruits and vegetables. Also, annual household income was associated with meeting the recommended intake for fruits and vegetables, and for red/processed meat. Poor physical functioning and sleep disturbance were significantly associated with meeting only the recommendation for red/processed meat. A similar study by Parker et al. (2014), which enrolled 31 AA BCSs, showed that most women met the dietary recommendations for fruits and vegetables (70%) and red meat (84%), but failed to meet the recommended intakes for fat, saturated fat, whole grains, added sugars, or total water. Wayne et al. (2006) demonstrated that better scores of physical functioning, body pain, social functioning, role-emotional, and mental health were associated with higher diet quality. Among BCSs, adherence to dietary recommendations is associated with lower recurrence and all-cause mortality (Inoue-Choi et al., 2013; Kwan et al., 2009), and increased intake of fruits and vegetables improves survival (Pierce et al., 2007). Among BCSs, improved diet quality promotes favorable nutrition-related biomarkers and healthy body weight (Pekmezi et al., 2011), and obesity may increase risk of cancer recurrence and comorbidities, such as cardiovascular disease and diabetes, and worsen overall survival (Protani et al., 2010). Suggested mechanisms of the association between body weight and cancer outcomes include alterations in circulating hormones, genomic instability, dysregulated growth signaling and cellular energetics, inhibition of apoptosis and immune surveillance, angiogenesis, insulin and insulin-like growth factor-1 signaling, and inflammatory modulation by adipokines (Demark-Wahnefried et al., 2012). Programs that enhance consumption of recommended diets should be part of management of breast cancer survivorship and support.

This study is among the few to examine adherence to dietary intake guidelines among AA BCSs, a group that is disproportionately disadvantaged by breast cancer morbidity and mortality. The limitations include recall bias resulting from the use of self-reported surveys. The lifestyle assessment tool used for the survey, however was developed from instruments that have been used for very large studies and have high validity and reliability scores. The small sample size of participants does not allow the results of this study to be generalized to other AA populations.

## CONCLUSIONS

Most AA BCSs are not meeting the guidelines on nutrition for cancer prevention, although improved diet quality promotes healthy body weight and survival among BCSs. Additional interventions that enhance access to and consumption of healthy diets among AA BCSs are needed.

## Figures and Tables

**Table 1:** Participant characteristics (N = 240)

	N (%)
**Age in years (mean ± SD)**	56.9 ± 11.8
< 50	61 (25.4)
50–64	118 (49.2)
≥ 65	44 (18.3)
Missing	17 (7.1)
**Race/Ethnicity**	
Black, non-Hispanic	219 (91.3)
Other	16 (6.6)
Missing	5 (2.1)
**Education**	
Less than college	64 (26.7)
Some college or above	172 (71.7)
*Missing*	*4 (1.7)*
**Employment**	
Employed	120 (50.0)
Unemployed	44 (18.4)
Retired	70 (29.2)
*Missing*	*6 (2.5)*
**Annual household income**	
< $25,000	68 (28.3)
$25,000 - $49,999	88 (36.7)
≥ $50,000	77 (32.1)
*Missing*	*7 (2.9)*
**Marital Status**	
Married	98 (40.8)
Single	52 (21.7)
Divorced/Widowed	83 (34.6)
*Missing*	*7 (2.9)*
**Meeting fruit and vegetable guidelines**	
Meet	4 (1.7)
Partially Meet	42 (17.5)
Not Meet	189 (78.8)
*Missing*	*5 (2.1)*
**Meeting red and processed meat guidelines**	
Meet	191 (95.4)
Partially Meet	30 (12.5)
Not Meet	8 (3.3)
*Missing*	*11 (4.6)*

**Table 2:** Bivariate analysis of participants meeting, partially meeting, or not-meeting the guideline for fruit and vegetable consumption and selected characteristics

	Fruit and vegetable guidelines			p-value
	Meet (n = 4)	Partially Meet (n = 42)	Not Meet (n = 189)	
Demographic	n (%)	n (%)	n (%)	
**Age in years (mean ± SD)**		*missing = 1*	*missing = 13*	0.200
< 50	2 (50.0)	8 (19.5)	50 (28.4)	
50–64	1 (25.0)	28 (68.3)	88 (50.0)	
≥ 65	1 (25.0)	5 (12.2)	38 (21.6)	
**Education**			*missing = 2*	0.403
Less than college	0 (0.0)	13 (31.0)	50 (26.7)	
Some college or above	4 (100.0)	29 (69.0)	137 (73.3)	
**Employment**			*missing = 4*	0.554
Employed	3 (75.0)	23 (54.8)	92 (49.7)	
Unemployed	1 (25.0)	9 (21.4)	33 (17.8)	
Retired	0 (0.0)	10 (23.8)	60 (32.4)	
**Annual household income**			*missing = 5*	0.100
< $25,000	1 (25.0)	9 (21.4)	57 (31.0)	
$25,000 - $49,999	0 (0.0)	14 (33.3)	73 (39.7)	
≥ $50,000	3 (75.0)	19 45.2)	54 (29.3)	
**Marital Status**			*missing = 5*	0.163
Married	4 (100.0)	18 (42.9)	74 (40.2)	
Single	0 (0.0)	11 (26.2)	40 (21.7)	
Divorced/Widowed	0 (0.0)	13 (31.0)	70 (38.0)	
**Clinical Characteristics**				
**Year since diagnosis**		*missing = 3*	*missing = 10*	0.749
< 5	1 (25.0)	10 (25.6)	42 (23.5)	
5–10	3 (75.0)	18 (46.2)	84 (46.9)	
> 10	0 (0.0)	11 (28.2)	53 (29.6)	
**Breast Cancer Recurrence**		*missing = 1*	*missing = 5*	0.508
Yes	0 (0.0)	8 (19.0)	42 (22.2)	
No	4 (100.0)	33 (78.6)	142 (75.1)	
**Surgery**				0.085
Yes	2 (50.0)	38 (90.5)	161 (85.2)	
No	2 (50.0)	4 (9.5)	28 (14.8)	
**Chemotherapy**				0.728
Yes	2 (50.0)	26 (61.9)	105 (55.6)	
No	2 (50.0)	16 (38.1)	84 (44.4)	
**Hormone treatments**				0.958
Yes	1 (25.0)	12 (28.6)	57 (30.2)	
No	3 (75.0)	30 (71.4)	132 (69.8)	
**Bone marrow/Stem cell transplant**				0.609
Yes	0 (0.0)	0 (0.0)	4 (2.1)	
No	4 (100.0)	42 (100.0)	185 (97.9)	
**Body Weight**				
**BMI (kg/m^2^)**	*missing = 1*	*missing = 6*	*missing = 32*	0.869
Healthy weight (<25)	1 (33.3)	10 (27.8)	43 (27.4)	
Overweight (25–29)	1 (33.3)	13 (36.1)	44 (28.0)	
Obese (≥30)	1 (33.3)	13 (36.1)	70 (44.6)	
**Post-diagnosis weight gain (lbs)**	*missing = 3*	*missing = 21*	*missing = 93*	0.483
< 20	1 (100.0)	6 (28.6)	41 (42.7)	
20 – 39	0 (0.0)	11 (52.4)	35 (36.5)	
≥ 40	0 (0.0)	4 (19.0)	20 (20.8)	
**Health-related quality of life score**				
**Physical functioning**			*missing = 1*	0.282
Poor	4 (100.0)	30 (71.4)	150 (79.8)	
Good	0 (0.0)	12 (28.6)	38 (20.2)	
**Anxiety**				0.713
Poor	0 (0.0)	4 (9.5)	13 (6.9)	
Good	4 (100.0)	38 (90.5)	176 (93.1)	
**Depression**		*missing = 1*	*missing = 1*	0.904
Poor	0 (0.0)	2 (4.9)	9 (4.8)	
Good	4 (100.0)	39 (95.1)	179 (95.2)	
**Fatigue**				0.722
Poor	0 (0.0)	6 (14.3)	25 (13.2)	
Good	4 (100.0)	36 (85.7)	164 (86.8)	
**Sleep disturbance**		*missing = 1*	*missing = 1*	0.539
Poor	0 (0.0)	6 (14.6)	35 (18.6)	
Good	4 (100.0)	35 (85.4)	153 (81.4)	
**Satisfaction with social role**			*missing = 1*	0.138
Poor	0 (0.0)	13 (31.0)	36 (19.1)	
Good	4 (100.0)	29 (69.0)	152 (80.9)	
**Pain interference**				0.677
Poor	0 (0.0)	6 (14.3)	22 (11.6)	
Good	4 (100.0)	36 (85.7)	167 (88.4)	
**Pain intensity**	*missing = 1*	*missing = 1*	*missing = 4*	0.513
Poor	1 (33.3)	15 (36.6)	85 (45.9)	
Good	2 (66.7)	26 (63.4)	100 (54.1)	

Note: p-value <0.05 is statistically significant

**Table 3:** Bivariate analysis of participants meeting, partially meeting, or not-meeting guideline for consumption of red and processed meat and selected characteristics

	Guidelines for red and processed meat			p-value
	Meet (n = 191)	Partially Meet (n = 30)	Not Meet (n = 8)	
Demographic	n (%)	n (%)	n (%)	
**Age in years (mean ± SD)**	*missing = 10*	*missing = 4*		0.655
< 50	48 (26.5)	7 (26.9)	4 (50.0)	
50–64	97 (53.6)	15 (57.7)	3 (27.5)	
≥ 65	36 (19.9)	4 (15.4)	1 (12.5)	
**Education**	*missing = 2*			0.048*
Less than college	44 (23.3)	12 (40.0)	4 (50.0)	
Some college or above	145 (76.7)	18 (60.0)	4 (50.0)	
**Employment**	*missing = 4*			0.324
Employed	94 (50.3)	19 (63.3)	4 (50.0)	
Unemployed	33 (17.6)	5 (16.7)	3 (37.5)	
Retired	60 (32.1)	6 (20.0)	1 (12.5)	
**Annual household income**	*missing = 5*			0.026*
< $25,000	47 (25.3)	12 (40.0)	3 (37.5)	
$25,000 - $49,999	70 (37.6)	15 (50.0)	1 (12.5)	
≥ $50,000	69 (37.1)	3 (10.0)	4 (50.0)	
**Marital Status**	*missing = 5*			0.040*
Married	80 (43.0)	11 (36.7)	4 (50.0)	
Single	35 (18.8)	13 (43.3)	2 (25.0)	
Divorced/Widowed	71 (38.2)	6 (20.0)	2 (25.0)	
**Clinical Characteristics**				
**Year since diagnosis**				0.167
< 5	42 (23.5)	8 (27.6)	3 (37.5)	
5–10	85 (47.5)	9 (31.0)	5 (62.5)	
> 10	52 (29.1)	12 (41.4)	0 (0.0)	
**Breast Cancer Recurrence**	*missing = 3*	*missing = 2*	*missing = 1*	0.063
Yes	37 (19.7)	11 (39.3)	2 (28.6)	
No	151 (80.3)	17 (60.7)	5 (71.4)	
**Surgery**				0.546
Yes	163 (85.3)	27 (90.0)	6 (75.0)	
No	28 (14.7)	3 (10.0)	2 (25.0)	
**Chemotherapy**				0.430
Yes	104 (54.5)	20 (66.7)	4 (50.0)	
No	87 (45.5)	10 (33.3)	4 (50.0)	
**Hormone treatments**				0.671
Yes	59 (30.9)	7 (23.3)	2 (25.0)	
No	132 (69.1)	23 (76.7)	6 (75.0)	
**Bone marrow/Stem cell transplant**				0.667
Yes	4 (2.1)	0 (0.0)	0 (0.0)	
No	187 (97.9)	30 (100.0)	8 (100.0)	
**Body Weight**				
**BMI (kg/m^2^)**	*missing = 31*	*missing = 4*	*missing = 2*	0.093
Healthy weight (<25)	48 (30.0)	3 (11.5)	2 (33.3)	
Overweight (25–29)	45 (28.1)	12 (46.2)	0 (0.0)	
Obese (≥30)	67 (41.9)	11 (42.3)	4 (66.7)	
**Post-diagnosis weight gain (lbs)**	*missing = 98*	*missing = 10*	*missing = 4*	0.228
< 20	42 (45.2)	4 (20.0)	2 (50.0)	
20 – 39	35 (37.6)	9 (45.0)	1 (25.0)	
≥ 40	16 (17.2)	7 (35.0)	1 (25.0)	
**Health-related quality of life score**				
**Physical functioning**	*missing = 1*			0.192
Poor	154 (81.1)	20 (66.7)	6 (75.0)	
Good	36 (18.9)	10 (33.3)	2 (25.0)	
**Anxiety**				0.609
Poor	13 (6.8)	1 (3.3)	1 (12.5)	
Good	178 (93.2)	29 (96.7)	7 (87.5)	
**Depression**				0.261
Poor	9 (4.8)	0 (0.0)	1 (12.5)	
Good	180 (95.2)	30 (100.0)	7 (87.5)	
**Fatigue**				0.894
Poor	25 (13.1)	3 (10.0)	1 (12.5)	
Good	166 (86.9)	27 (90.0)	7 (87.5)	
**Sleep disturbance**	*missing = 1*			0.205
Poor	36 (18.9)	3 (10.0)	0 (0.0)	
Good	154 (81.1)	27 (90.0)	8 (100.0)	
**Satisfaction with social role**	*missing = 1*			0.786
Poor	38 (20.0)	7 (23.3)	1 (12.5)	
Good	152 (80.0)	23 (76.7)	7 (87.5)	
**Pain interference**				0.348
Poor	23 (12.0)	2 (6.7)	2 (25.0)	
Good	168 (88.0)	28 (93.3)	6 (75.0)	
**Pain intensity**	*missing = 5*			0.049*
Poor	88 (47.3)	7 (23.3)	3 (42.9)	
Good	98 (52.7)	23 (76.7)	4 (57.1)	

* p-value <0.05 is statistically significant

**Table 4:** Regression analyses of factors related to meeting the fruit and vegetable guideline by BCSs

	Fruit and vegetable model *Meet/Partially Meet vs. Not Meet (reference)*		
Variables	OR	95% CI	*P*
**Age in years**			
< 50	0.829	0.205, 3.348	0.792
50–64	1.490	0.452, 4.914	0.513
≥ 65	Ref		
**Education**			
Less than college	1.919	0.758, 4.860	0.169
Some college or above	ref		
**Employment**			
Employed	1.401	0.504, 3.898	0.518
Unemployed	1.997	0.578, 6.904	0.274
Retired	ref		
**Annual household income**			
< $25,000	0.246	0.075, 0.801	0.020*
$25,000 - $49,999	0.318	0.131, 0.774	0.012*
≥ $50,000	ref		
**Marital Status**			
Married	1.058	0.445, 2.515	0.899
Single	1.372	0.514, 3.658	0.528
Divorced/Widowed	ref		
**Surgery**			
No	0.817	0.301, 2.217	0.691
Yes	ref		

* p-value <0.05 is statistically significant

**Table 5:** Regression analyses of factors related to meeting the guideline for consumption of red and processed meat guideline by BCSs

	Red and processed meat Model *Meet vs. Partially Meet/Not Meet (reference)*		
Variables	OR	95% CI	*p*
**Age in years**			
< 50	1.146	0.017, 75.258	0.949
50–64	5.089	0.111, 232.374	0.404
≥ 65	Ref		
**Education**			
Less than college	0.240	0.041, 1.394	0.112
Some college or above	Ref		
**Employment**			
Employed	0.021	0.000, 1.902	0.093
Unemployed	0.895	0.021, 37.459	0.954
Retired	ref		
**Annual household income**			
< $25,000	0.020	0.000, 0.888	0.043*
$25,000 - $49,999	0.008	0.000, 0.513	0.023*
≥ $50,000	ref		
**Marital Status**			
Married	0.319	0.032, 3.165	0.329
Single	0.182	0.020, 1.673	0.132
Divorced/Widowed	ref		
**Year since diagnosis**			
< 5	0.427	0.026, 7.098	0.553
5–10	0.642	0.089, 4.630	0.660
> 10	ref		
**Breast Cancer Recurrence**			
Yes	0.116	0.013, 1.070	0.057
No	ref		
**BMI (kg/m^2^)**			
Healthy weight (<25)	2.077	0.088, 48.994	0.650
Overweight (25–29)	1.188	0.145, 9.768	0.873
Obese (≥30)	ref		
**Post-diagnosis weight gain (lbs)**			
< 20	0.150	0.007, 3.040	0.217
20 – 39	2.147	0.276, 16.711	0.465
≥ 40	ref		
**Physical functioning**			
Poor	38.481	2.255, 656.579	0.012*
Good	ref		
**Sleep disturbance**			
Poor	60.841	1.612, 2296.022	0.027*
Good	ref		
**Pain intensity**			
Poor	0.536	0.074, 3.910	0.539
Good	ref		

* p-value <0.05 is statistically significant

## Statement of Student-Mentor Relationship

The lead author for this report, Lindsey A. Ramirez, a Master of Science in Psychology student, participated in the Summer Public Health Scholars Program in the Institute of Public and Preventive Health at Augusta University. Dr. Selina A. Smith, the senior author, served as her mentor.
